# Duplicate prescriptions in the emergency department: a retrospective cohort study

**DOI:** 10.1007/s00228-022-03436-6

**Published:** 2022-12-08

**Authors:** Johannes Heck, Benjamin Krichevsky, Adrian Groh, Martin Schulze Westhoff, Hans Laser, Swetlana Gerbel, Patrick-Pascal Strunz, Carsten Schumacher, Martin Klietz, Dirk O. Stichtenoth, Christoph Höner zu Siederdissen, Olaf Krause

**Affiliations:** 1 Institute for Clinical Pharmacology, Hannover Medical School, Carl-Neuberg-Str. 1, 30625 Hannover, Germany; 2 Institute for General Practice and Palliative Care, Hannover Medical School, Hannover, Germany; 3 Medical Service of the German Armed Forces, Kiel, Germany; 4 Department of Psychiatry, Social Psychiatry and Psychotherapy, Hannover Medical School, Hannover, Germany; 5 Center for Information Management, Hannover Medical School, Hannover, Germany; 6 Department of Internal Medicine II, Rheumatology and Clinical Immunology, University Hospital Würzburg, Würzburg, Germany; 7 Center for Clinical Trials, Hannover Medical School, Hannover, Germany; 8 Department of Neurology, Hannover Medical School, Hannover, Germany; 9 Drug Commissioner, Hannover Medical School, Hannover, Germany; 10 Emergency Department, Hannover Medical School, Hannover, Germany; 11 Center for Medicine of the Elderly, DIAKOVERE Henriettenstift, Hannover, Germany

**Keywords:** Duplicate prescriptions, Medication review, Pharmacotherapy safety, Emergency department, Internal medicine

## Abstract

**Purpose:**

To determine the nature and frequency of duplicate prescriptions (DPs) in the emergency department (ED) by utilization of a novel categorization of DPs which differentiates between appropriate DPs (ADPs) and potentially inappropriate DPs (PIDPs).

**Methods:**

In this retrospective cohort study, adult patients who presented to the ED for internal medicine of a large university hospital in northern Germany in 2018 and 2019 were screened for the presence of DPs. Descriptive statistical methods were used to characterize the nature and frequency of PIDPs compared to the frequency of ADPs.

**Results:**

A total of 4208 patients were enrolled into the study. The median age of the study population was 63 years (interquartile range (IQR) 48–77), 53.9% were female. The patients took a median of 5 drugs (IQR 3–9). 10.9% of the study population were affected by at least one PIDP (at least one grade-1 PIDP: 6.1%; at least one grade-2 PIDP: 4.5%; at least one grade-3 PIDP: 1.1%). Non-opioid analgesics accounted for the majority of grade-1 PIDPs, while inhalatives were most frequently responsible for grade-2 and grade-3 PIDPs. Nearly half of the study population (48.6%) displayed at least one ADP.

**Conclusion:**

PIDPs pose a frequent pharmacological challenge in the ED. The medication review should comprise a systematic screening for PIDPs with a particular focus on non-opioid analgesics and inhalatives. ADPs were detected more frequently than PIDPs, questioning the predominant notion in the medical literature that DPs are exclusively deleterious.

**Supplementary Information:**

The online version contains supplementary material available at 10.1007/s00228-022-03436-6.

## Introduction

Adverse drug reactions (ADRs) account for approximately 5 to 7% of presentations to the emergency department (ED) [1–5] and constitute a frequent cause of hospitalization [6], especially in the elderly population [7]. Inappropriate prescribing has been recognized as a risk factor for ADR occurrence [8], and duplicate prescriptions (DPs) are considered as an important contributor to inappropriate prescribing [9]. In the scientific literature, a plethora of definitions of DPs exist. However, the majority of these definitions have been devised by pharmacists to investigate pharmaceutical interventions [9–16], and are therefore of limited utility to physicians in medical settings. In addition, most of the existing definitions view DPs exclusively as detrimental to prescribing quality and to patient safety, neglecting the fact that many DPs—such as the co-administration of two platelet aggregation inhibitors following coronary stent implantation (e.g. low-dose acetylsalicylic acid plus a P2Y_12_ receptor antagonist)—are supported by evidence-based guidelines [17] and are routinely used in clinical practice.

The inconsistency, limited clinical utility, and lacking differentiation between therapeutically desired and undesired DPs of the definitions currently prevailing in the medical literature represent major obstacles to the comparability of studies in the field. To overcome these limitations, we recently proposed a new categorization of DPs that strongly emphasizes the physician’s view [18]. First and foremost, we suggest the differentiation between appropriate DPs (ADPs) and potentially inappropriate DPs (PIDPs) (Table 1). We deliberately chose the term PIDP with reference to the expression PIM (potentially inappropriate medication, i.e. a drug with an unfavorable benefit-to-risk profile in elderly people [19]), thereby highlighting the importance of pharmacotherapy safety within our terminology. PIDPs are further subdivided into three grades, with higher grades indicating an increasing degree of inappropriateness [18].

**Table 1:** Categorization of duplicate prescriptions (DPs) in appropriate duplicate prescriptions (ADPs) and potentially inappropriate duplicate prescriptions (PIDPs). PIDPs are further subdivided into three grades, with higher grades indicating an increasing degree of inappropriateness (adopted and modified from Heck et al. [18] under a Creative Commons Attribution 4.0 International License). Of note, the degree of inappropriateness does not necessarily correspond to the clinical severity of an adverse drug reaction that might result from a PIDP

	**Appropriate duplicate prescriptions (ADPs)**	**Potentially inappropriate duplicate prescriptions (PIDPs)**		
**Grade**	**—**	**1**	**2**	**3**
**Description**	Two drugs with therapeutically desired synergistic effects (i.e. established combination treatments)	Two drugs with overlapping or comparable pharmacodynamics	Two drugs of the same therapeutic class (i.e. targeting the same molecular structure)	Two times the same drug (exceeding the recommended maximum daily dose)
**Examples**	HMG-CoA reductase inhibitor + ezetimibe	ACE inhibitor + ARB	Two different opioid analgesics (e.g. buprenorphine + hydromorphone; oxycodone + tramadol)	Hydrochlorothiazide both as single agent and as partner in an antihypertensive combination product
	Metformin + SGLT2 inhibitor	PPI + H_2_ receptor antagonist	Hydrochlorothiazide + chlorthalidone	Valsartan both as single agent and in sacubitril–valsartan
	Opioid analgesic + non-opioid analgesic	Paracetamol + ibuprofen	Ibuprofen + diclofenac	Paracetamol both as single agent and in an acetylsalicylic acid–paracetamol–caffeine combination product
	Acetylsalicylic acid + clopidogrel	Ibuprofen + metamizole	Amlodipine + lercanidipine	Diclofenac both as single agent and in a diclofenac–misoprostol combination product
	Loop diuretic + thiazide diuretic	Doxylamine + zopiclone	Diphenhydramine + doxylamine	Codeine both as single agent and in a paracetamol–codeine combination product

*ACE* angiotensin-converting enzyme, *ARB* angiotensin-receptor blocker, *HMG-CoA* hydroxymethylglutaryl coenzyme A, *PPI* proton pump inhibitor, *SGLT2* sodium–glucose cotransporter type 2

The decision whether a DP is appropriate or potentially inappropriate is a challenging task which requires detailed knowledge about patient characteristics such as medical history, contraindications, and—last but not least—patient preferences [18]. Our DP categorization is therefore intended to be applied by physicians with direct patient contact, for example in the ED. It was not devised for patient counseling or drug dispensation at pharmacies.

Rapid recognition of potentially deleterious drug combinations is of pivotal importance in the ED; however, the prevalence of DPs in the ED has not been established to date. In this pilot study, we investigated the prevalence of ADPs and PIDPs in the ED for internal medicine of an approximately 1900-bed university hospital in northern Germany over the course of 2 years.

The primary goals of our study were to elucidate the nature and frequency of PIDPs, and to determine the ratio between ADPs and PIDPs as an indicator of prescribing quality. We are convinced that in-depth knowledge about the characteristics of PIDPs will increase emergency physicians’ awareness for pharmacotherapy safety and will contribute to the improvement of patient care.

## Methods

### Study design and setting

The present study was a retrospective pilot study conducted at Hannover Medical School, a large university hospital and tertiary care referral center in northern Germany, between 01 January 2018 and 31 December 2019. The study period 2018–2019 (i.e. two complete calendar years) was chosen to (i) preclude a possible influence of the coronavirus disease 2019 (COVID-19) pandemic on ED statistics (the first COVID-19 case in Germany was reported on 27 January 2020 [20]) and (ii) to diminish confounding of the study results by seasonal fluctuations (e.g. increased use of anti-allergic drugs during spring months). We used the Strengthening the Reporting of Observational Studies in Epidemiology (STROBE) cohort checklist when writing our report [21].

### Eligibility criteria

Patients were eligible for enrolment in the study (i) if they presented to the ED for internal medicine of Hannover Medical School between 01 January 2018 and 31 December 2019, (ii) if they were ≥ 18 years of age, (iii) if their medication was documented electronically, and (iv) if they were taking ≥ 2 drugs (prescription medicines, over-the-counter (OTC) drugs as well as complementary and alternative medicines (CAMs), taken regularly or on an as-needed basis). All patients or their legal representatives had provided written informed consent that patient-related data be used for research purposes.

### Patient selection and data acquisition

The study population was identified and medical data (demographic characteristics, anamnesis, International Statistical Classification of Diseases and Related Health Problems 10^th^ Revision (ICD-10) diagnoses, and currently taken medications (as documented by treating physicians upon patient contact in the ED)) were made available in a structured format by the Enterprise Clinical Research Data Warehouse (ECRDW). The ECRDW is one of the largest medical data repositories worldwide and is maintained by the Center for Information Management of Hannover Medical School [22].

### Assessment of medication classes

Drugs taken in the study population were grouped into 58 different medication classes (Supplementary Table 1). The applied medication classification system is based on the World Health Organization’s Anatomical Therapeutic Chemical (ATC) system [23], but primarily takes the pharmacotherapeutic perspectives of physicians into consideration. In our experience, physicians do not operate with ATC codes in clinical routine. Therefore, the study team opted for a more clinically oriented and human readable modification of the ATC system for the purpose of this study.

### Definition of appropriate duplicate prescriptions and potentially inappropriate duplicate prescriptions

ADPs and PIDPs were defined according to the recently proposed DP categorization by Heck et al. [18] which is outlined in Table 1. ADPs are rational and established combination treatments, in contrast to PIDPs. PIDPs are subdivided into three different grades, with higher grades indicating an increasing degree of inappropriateness. It must be noted, however, that the degree of inappropriateness does not necessarily reflect clinical severity; hence, it is possible that an ADR resulting from a grade-1 PIDP is clinically more severe than an ADR resulting from a grade-2 or grade-3 PIDP. Importantly, all decisions about appropriateness/inappropriateness of DPs were made on a case-by-case analysis, diligently evaluating medication regimens in view of patients’ anamnesis, age, sex, and comorbidities. The definitions and examples displayed in Table 1 served as a theoretical basis. However, the ultimate decision whether a specific DP was considered appropriate or potentially inappropriate (and if so, which grade of inappropriateness) was made individually in each case.

### Outcome measures

The outcome measures of this study were the nature and frequency of PIDPs in adult patients presenting to the ED for internal medicine, stratified by age groups and compared with the frequency of ADPs.

### Identification of appropriate duplicate prescriptions and potentially inappropriate duplicate prescriptions

Patient records created at presentation to the ED were manually screened for ADPs and PIDPs by two investigators (JH and BK). JH and BK, who are specialized in clinical pharmacology and internal medicine, respectively, analyzed all patient records together as a team. This approach was considered most suitable for the purpose of this study as it safeguarded that each case was evaluated both from a clinical-pharmacological perspective and simultaneously from the point of view of an internist. JH and BK reached a common decision for each case by means of discussion and consensus. Interrater reliability was not formally evaluated. All identified ADPs and PIDPs were validated by an interdisciplinary expert panel (OK, CHzS, PPS: specialists in internal medicine; OK, AG: specialists in geriatric medicine; CHzS, CS, OK: specialists in emergency medicine; CS: specialist in anesthesiology and intensive care medicine; AG, MSW: specialists in psychiatry and psychopharmacology; MK: specialist in neurology; DOS: specialist in clinical pharmacology and Drug Commissioner of Hannover Medical School). The cases were distributed among the panelists based on their professional expertise. The panelists validated ADPs and PIDPs independently. Of note, the panelists had access to the same patient records and to the same amount of clinical information as the primary investigators (i.e. JH and BK) and, therefore, the panelists also made their decisions for each patient individually.

### Statistical analysis

Descriptive statistical techniques were used to summarize the data. Patient characteristics are presented as absolute and relative frequencies for categorical variables and as medians with interquartile ranges (IQRs) for quantitative variables due to skewed distribution. Frequencies were explored in the entire study population and in subgroups. Specifically, we compared patients affected by at least one PIDP (hereon referred to as patients with PIDPs) with patients not affected by PIDPs (hereon referred to as patients without PIDPs). Differences between patients with and without PIDPs were analyzed with Pearson’s chi-squared test for categorical variables (i.e. variables “age group”, “sex”, “presence of polypharmacy”, and “presence of ADPs”) and Mann–Whitney *U* test for quantitative variables (i.e. variables “age” and “number of drugs taken”) [24]. Fundamentally, *P* values (two-sided) < 0.05 were considered as statistically significant. To reduce the number of type I errors owing to multiple testing, an adapted Bonferroni correction was applied [25]. The variables “age group” and “polypharmacy” were derived from the variables “age” and “number of drugs taken”, respectively. Therefore, the level of significance (i.e. 0.05) was divided by 4 instead of 6 in the Bonferroni correction, yielding an adjusted level of significance of 0.05 ÷ 4 = 0.0125. Hence, *P* values (two-sided) < 0.0125 were considered as statistically significant. This statistical approach was considered as most appropriate for the purpose of this study since on the one hand it addressed the problem of multiple testing, while on the other hand it was not overly conservative [25]. Further details on the statistical methods are specified in Table 2. All statistical analyses were performed with IBM^®^ SPSS^®^ Statistics 28 (Armonk, New York, USA).

**Table 2:** Characteristics of the study population

**Characteristic and category**	**Total**	**Patients without PIDPs**	**Patients with PIDPs**	***P*** **value**
	**(*n*** = **4208)**	**(*n*** = **3750; 89.1%)**	**(*n*** = **458; 10.9%)**	
Median age (IQR)—years	63 (48–77)	63 (49–77)	60 (44–76)	**0.008**^**a**^
Age group—% (no.)				**0.011**^**b**^
18–29 years	8.2 (346)	7.8 (293)	11.6 (53)	
30–39 years	7.9 (333)	7.8 (292)	9.0 (41)	
40–49 years	10.9 (458)	10.6 (397)	13.3 (61)	
50–59 years	17.3 (728)	17.5 (656)	15.7 (72)	
60–69 years	17.7 (746)	18.2 (683)	13.8 (63)	
70–79 years	19.9 (838)	19.8 (744)	20.5 (94)	
80–89 years	14.6 (613)	14.9 (557)	12.2 (56)	
≥ 90 years	3.5 (146)	3.4 (128)	3.9 (18)	
Female sex—% (no.)	53.9 (2268)	53.5 (2005)	57.4 (263)	0.109^b^
Median number of drugs (IQR)	5 (3–9)	5 (3–8)	8 (5–12)	** < 0.001**^**a**^
Polypharmacy—% (no.)	58.2 (2450)	56.0 (2101)	76.2 (349)	** < 0.001**^**b**^
Patients with ADPs—% (no.)	48.6 (2045)	49.3 (1849)	42.8 (196)	**0.008**^**b**^

Patients with PIDPs were defined as patients affected by at least one PIDP. Patients without PIDPs were defined as patients not affected by PIDPs. Patients with ADPs were defined as patients who displayed at least one ADP. Polypharmacy was defined as the concomitant intake of ≥ 5 drugs

*ADP* appropriate duplicate prescription, *IQR* interquartile range, *PIDP* potentially inappropriate duplicate prescription

^a^Quantitative variables were tested for normality by utilization of the Shapiro–Wilk test and by inspection of the histogram and Q–Q plot. Since the data did not follow a normal distribution, differences between patients with and without PIDPs were compared with the Mann–Whitney *U* test as a nonparametric test [24]. Significant *P* values are highlighted in bold

^b^Pearson’s chi-squared test was used to assess whether the presence of PIDPs was related to age groups, sex, presence of polypharmacy, or the presence of ADPs. Significant *P* values are highlighted in bold

## Results

### Study population

**Fig. 1:**
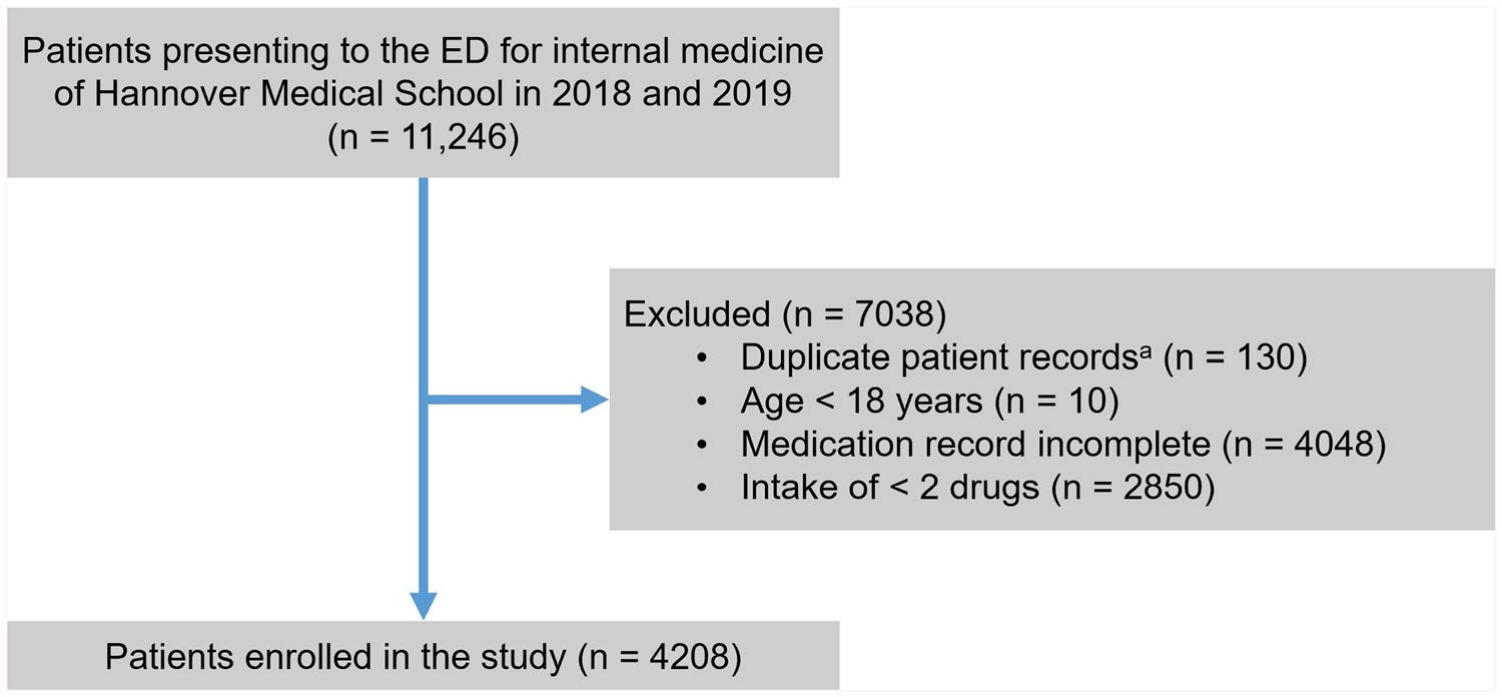
Flow of participants. ^a^Duplicate patient records were records that had been created twice, e.g. by two different physicians who separately examined the patient in the ED. Of these duplicates, the version containing less clinical information was excluded. ED denotes emergency department

Overall, 11,246 patients were identified by ECRDW and were manually screened for eligibility by the joint first authors (JH and BK). Of these 11,246 patients, 4208 (37.4%) fulfilled the inclusion criteria and were enrolled in the study. The reasons for exclusion of the patients who did not meet the eligibility criteria are depicted in Fig. 1. The most frequent reason for exclusion from the study was incompleteness of the medication record (36.0%; 4048/11,246).

The median age of the study population was 63 years (IQR 48–77 years; range 18–103 years) and 53.9% (2268/4208) of the study population were female (Table 2). The patients took a median of 5 drugs (IQR 3–9 drugs; range 2–32 drugs). Overall, 26,402 drugs were taken in the study population, with vitamins/hormones/enzymes/growth factors/minerals/trace elements (13.0%; 3441/26,402), beta-blockers (6.9%; 1821/26,402), proton pump inhibitors (6.3%; 1656/26,402), platelet aggregation inhibitors (5.2%; 1372/26,402), and hydroxymethylglutaryl coenzyme A (HMG-CoA) reductase inhibitors (statins) (5.0%; 1331/26,402) representing the five most frequently used medication classes (Supplementary Table 1).

### Potentially inappropriate duplicate prescriptions

**Fig. 2:**
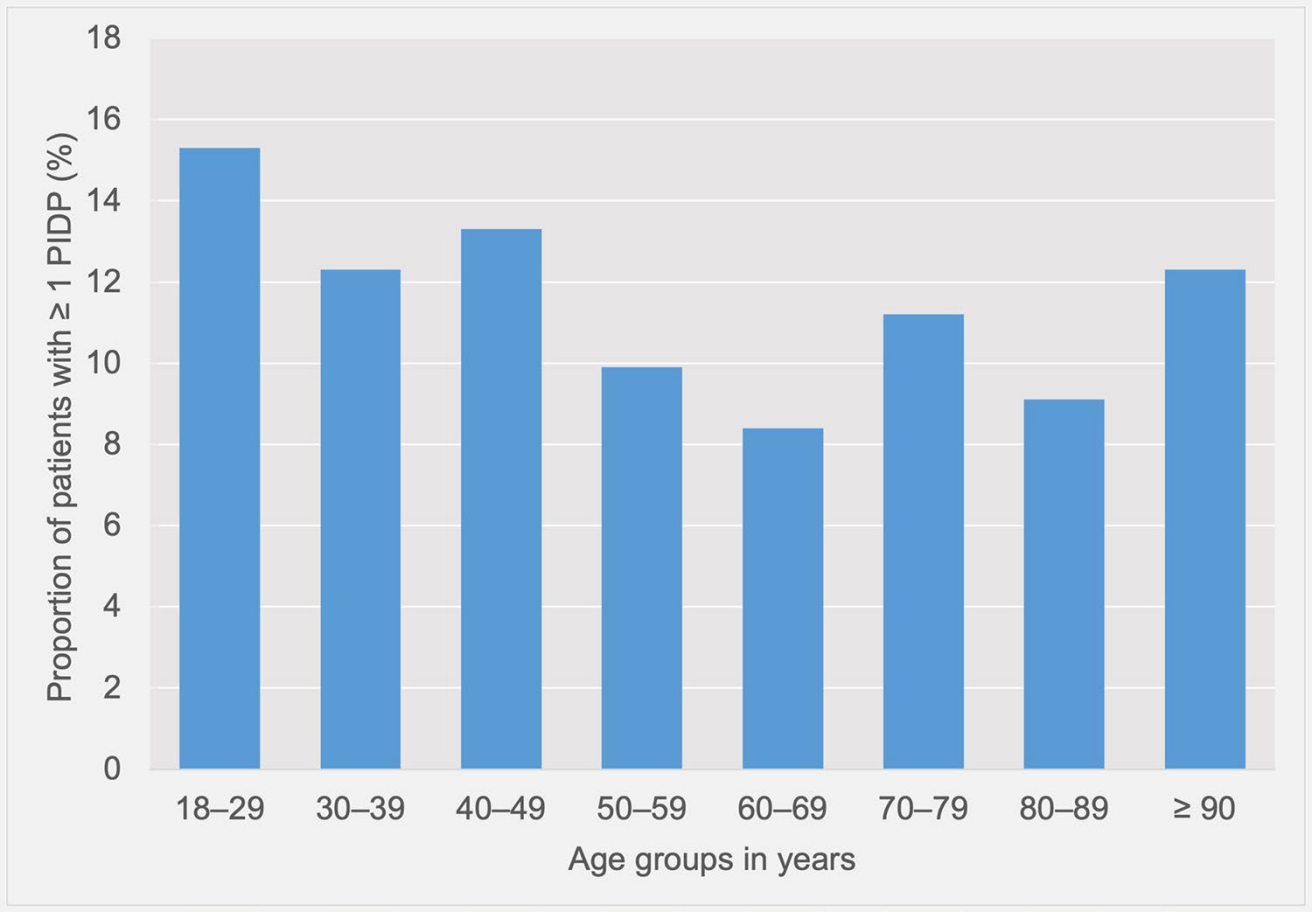
Proportion of patients affected by at least one potentially inappropriate duplicate prescription (PIDP), stratified by age groups

10.9% of the study population (458/4208) were affected by at least one PIDP. As demonstrated in Table 2, patients with PIDPs were significantly younger than patients without PIDPs (median 60 years (IQR 44–76 years) vs. 63 years (IQR 49–77 years); *P* = 0.008). The proportion of patients with PIDPs stratified by age groups is displayed in Fig. 2. Remarkably, we detected the highest proportion of patients with PIDPs in patients aged 18–29 years (15.3%; 53/346) and the lowest proportion in the age group of 60–69 years (8.4%; 63/746).

With respect to PIDP grades, 6.1% of the study population (258/4208) were affected by at least one grade-1 PIDP, 4.5% (190/4208) by at least one grade-2 PIDP, and 1.1% (45/4208) by at least one grade-3 PIDP.

**Fig. 3:**
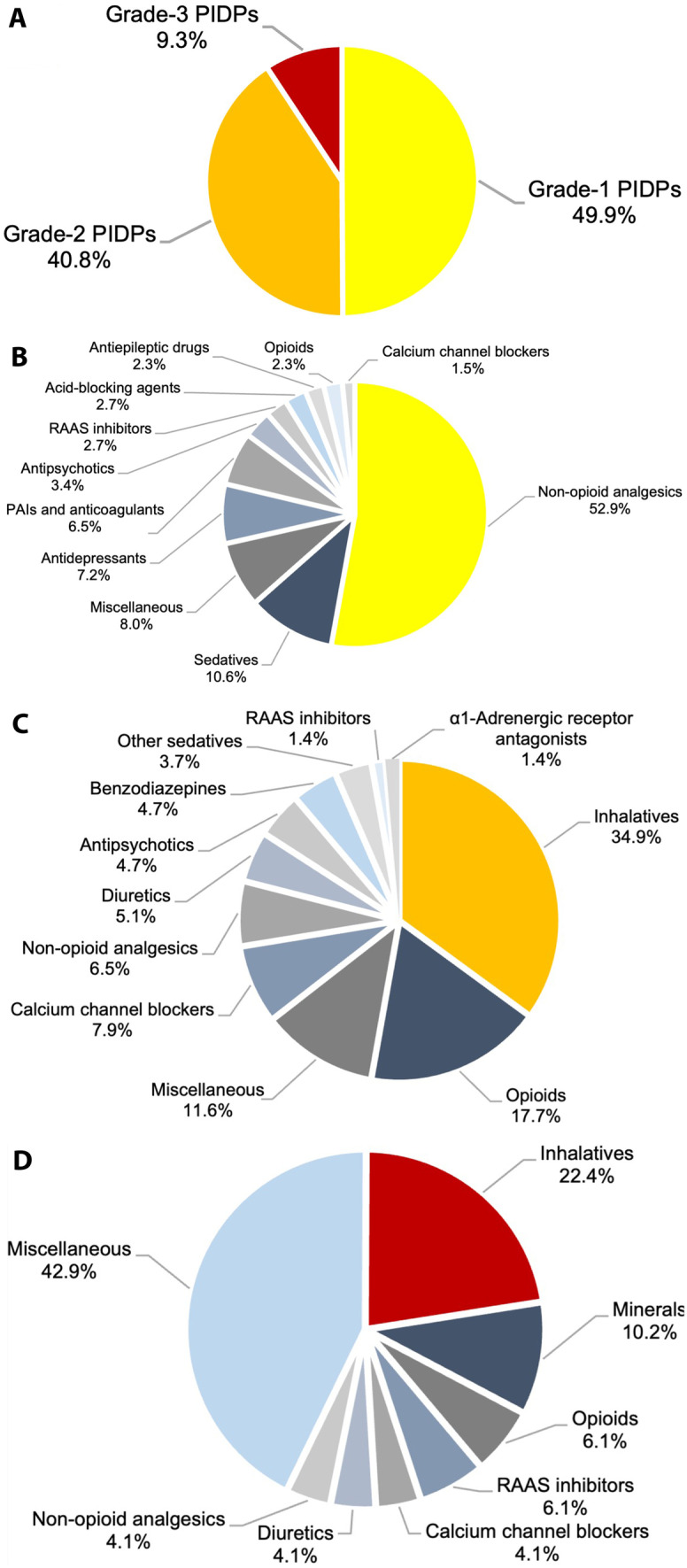
Subcategorization of potentially inappropriate duplicate prescriptions (*n* = 527) detected in the study population. **A** Distribution of PIDPs into grades 1 to 3. **B** Medication classes involved in grade-1 PIDPs (n = 263). **C** Medication classes involved in grade-2 PIDPs (*n* = 215). **D** Medication classes involved in grade-3 PIDPs (*n* = 49). PAI denotes platelet aggregation inhibitor, PIDP potentially inappropriate duplicate prescription, and RAAS renin–angiotensin–aldosterone system

Overall, 527 PIDPs were detected in the study population. The distribution of PIDPs into grades 1 to 3 as well as the medication classes involved are showcased in Fig. 3. The absolute and relative frequencies of drug combinations implicated in PIDPs grades 1 to 3 are tabulated in Supplementary Table 2, while the absolute counts of patients affected by PIDPs grades 1 to 3—stratified by age groups and medication classes—are shown in Supplementary Tables 3A–C.

Non-opioid analgesics were most frequently responsible for grade-1 PIDPs (52.9%; 139/263), with ibuprofen + metamizole (40/139) and paracetamol + metamizole (37/139) being the predominant combinations. It must be noted that combinations of non-opioid analgesics were only considered as potentially inappropriate if the respective substances were co-administered at the same time point without further clinical differentiation. By contrast, if—for example—paracetamol and ibuprofen were administered in alternating order at different time points to counterbalance hepatic and renal ADRs in a patient, this regimen was considered appropriate, highlighting the importance of manual case assessment that was performed in this study. Of particular interest, non-opioid analgesics accounted for approximately 75% of grade-1 PIDPs (44/59) in patients below the age of 40 years. By contrast, sedatives were most frequently responsible for grade-1 PIDPs in patients aged between 70 and 89 years (17.3%; 14/81). Platelet aggregation inhibitors and anticoagulants accounted for 13.0% of grade-1 PIDPs (16/123) in patients 60 years of age or older.

Inhalatives were most frequently responsible for grade-2 PIDPs (34.9%; 75/215), with fenoterol + salbutamol (11/75) and formoterol + salbutamol (9/75) being most frequently implicated. Of note, the combination of a long- and a short-acting beta-2 agonist was only considered potentially inappropriate if both drugs were applied at the same time point on a regular basis. By contrast, the combination of a long-acting beta-2 agonist applied regularly and a short-acting beta-2 agonist inhaled on an as-needed basis was considered appropriate. Opioid analgesics accounted for 17.7% of grade-2 PIDPs (38/215), most notably the combination fentanyl + morphine (8/38).

Inhalatives were most frequently responsible for grade-3 PIDPs (22.4%; 11/49), most prominently the combination salbutamol + salbutamol (4/11). Of interest, inhalatives accounted for 29.0% of grade-3 PIDPs (9/31) in patients aged between 40 and 79 years. Remarkably, 42.9% of grade-3 PIDPs (21/49) could not be attributed to a distinct medication class (category “Miscellaneous” in Supplementary Table 2 and Fig. 3D), with cholecalciferol + cholecalciferol accounting for one third (7/21) of “miscellaneous” grade-3 PIDPs.

### Appropriate duplicate prescriptions

Nearly half of the study population (48.6%; 2045/4208) displayed at least one ADP (hereon referred to as patients with ADPs). Of particular interest, the proportion of patients with ADPs was markedly lower among patients with PIDPs than among patients without PIDPs (42.8% (196/458) vs. 49.3% (1849/3750); *P* = 0.008) (Table 2). The proportion of patients with ADPs was considerably higher among patients 60 years of age or older (55.9%; 1309/2343) compared to patients below the age of 60 (39.5%; 736/1865).

### Relationship between appropriate duplicate prescriptions and potentially inappropriate duplicate prescriptions

Astonishingly, the number of patients with ADPs but without PIDPs (*n* = 1849) was approximately 7.1 times higher than the number of patients with PIDPs but without ADPs (*n* = 262).

### Polypharmacy

Polypharmacy, defined as the simultaneous intake of ≥ 5 different drugs [26], was present in more than half of the study population (58.2%; 2450/4208). A higher proportion of patients with PIDPs were affected by polypharmacy compared to patients without PIDPs (76.2% (349/458) vs. 56.0% (2101/3750); *P* < 0.001). Correspondingly, patients with PIDPs took significantly more drugs than patients without PIDPs (median 8 drugs (IQR 5–12 drugs) vs. 5 drugs (IQR 3–8 drugs); *P* < 0.001).

## Discussion

The present study provides two main results. First, we detected that 10.9% of patients presenting to the ED were affected by PIDPs. 6.1%, 4.5%, and 1.1% of the study population were exposed to at least one grade-1, grade-2, and grade-3 PIDP, respectively. Non-opioid analgesics accounted for the majority of grade-1 PIDPs, while inhalatives were most frequently responsible for grade-2 and grade-3 PIDPs.

Second, the number of patients with ADPs but without PIDPs was more than 7 times higher than the number of patients with PIDPs but without ADPs. We propose the term ADP-to-PIDP ratio for this measure and we suggest that it might serve as a marker of prescribing quality in future pharmacoepidemiological studies, with higher values reflecting better prescribing quality. As a limitation, the ADP-to-PIDP ratio can only be interpreted meaningfully on an aggregated patient level. It must be taken into consideration that approximately 5% of the study population (196/4208) displayed both ADPs and PIDPs. Although challenging to interpret on the individual patient level, this constellation most likely corresponds to an intermediate prescribing quality. The ADP-to-PIDP ratio that we propose is focused on patients. Future studies might also investigate ratios that are centered on prescriptions, e.g. the number of ADPs in a study cohort divided by the total number of DPs, or the number of PIDPs divided by the total number of DPs.

Of note, the two key findings of our study are not mutually exclusive. Quite the opposite, they do complement each other: while PIDPs pose a frequent pharmacological challenge in the ED, our results indicate that the majority of DPs are actually appropriate. The high number of ADPs may reflect the trend to combination treatments in internal medicine, for example in the treatment of arterial hypertension [27], dyslipidemia [28], and type-2 diabetes mellitus [29].

In the following, we are going to compare our study results to other investigations in the field. We point out that owing to diverging terminology and different definitions of DPs (subsequently highlighted with quotation marks upon first appearance in the text), comparisons between our findings and results from other studies must be interpreted with circumspection.

Zwietering et al. investigated the prevalence of “double medications” in a cohort of 200 patients in a geriatric outpatient clinic in The Netherlands [30]. The prevalence of double medications was low, ranging from 0 to 1.9%, depending on the mode of the medication review [30]. Of note, the study cohort was considerably older than in our investigation (mean age (± standard deviation) 82 (± 6) years) vs. median age (IQR) 63 (48–77) years), suggesting that the presence of double medications/PIDPs is not associated with advanced age.

Kinoshita et al. detected a prevalence of “duplicative medications” of 8.8% among insurants of a corporate Japanese health insurance society [31]. It must be emphasized that the authors did not exclude minor patients from their study; therefore, the study population was markedly younger than in our analysis (mean age (± standard deviation) 33.4 (± 17.5) years vs. median age (IQR) 63 (48–77) years) [31]. Moreover, Kinoshita et al. examined health insurance data [31], while we focused on clinical data. Similar to Kinoshita et al. we detected the lowest prevalence of PIDPs in the age group of 60–69 years. By contrast, younger patients (0–29 years in the Kinoshita et al. study; 18–29 years in our study) were most frequently affected by duplicative medications/PIDPs [31]. We hypothesize that elderly people visit physicians more regularly than younger people and might thus have their medication regimens checked more frequently. Besides, medication reviews might be conducted more thoroughly in elderly patients because of age-associated multimorbidity and polypharmacy [8], leading to a higher detection and correction rate of PIDPs and a higher prevalence of ADPs among the elderly. In addition, we speculate that younger people might take OTC drugs such as non-opioid analgesics more frequently without medical supervision, resulting in a higher prevalence of PIDPs in this age group. Surprisingly, while antibiotics constituted a frequent cause of duplicative medications in the Kinoshita et al. study [31], they did not elicit any PIDPs in our study. This discrepancy might be explained by the fact that all DPs of antibiotics in our study were appropriate (e.g. the co-administration of trimethoprim and sulfamethoxazole), whereas Kinoshita et al. did not differentiate between appropriate and inappropriate DPs [31].

Comparable to Kinoshita et al., Heinze et al. also examined health insurance data and estimated that 13 to 15% of the Austrian population treated with antihypertensives, lipid-lowering agents, or antidiabetics were affected by “double medications” [32]. The authors conceded, however, that a differentiation between appropriate and inappropriate double medications was not possible with their methodology [32]. Moreover, the definition of double medications used by Heinze et al. precluded the detection of grade-3 PIDPs [32].

In a retrospective cohort study, Freytag et al. detected 2 “duplicate prescriptions” equivalent to grade-3 PIDPs among 3189 multimorbid elderly outpatients, both elicited by the co-administration of diclofenac + diclofenac [33]. Of the ten most frequently encountered combinations of analgesics, duplicate prescriptions equivalent to grade-1 PIDPs, grade-2 PIDPs, and ADPs accounted for 49.5%, 20.0%, and 30.5%, respectively [33]. Diclofenac + metamizole and diclofenac + ibuprofen were most frequently responsible for duplicate prescriptions equivalent to grade-1 and grade-2 PIDPs, respectively [33].

Similar to Freytag et al., Kovac et al. examined “dual use” of analgesics and demonstrated an association between dual nonsteroidal anti-inflammatory drug (NSAID) use and poorer physical health-related quality of life, albeit in a highly selected patient population [34]. All participants were NSAID users [34]. According to our DP categorization, dual NSAID use as defined by Kovac et al. is equivalent to PIDPs grade-2 (e.g. ibuprofen + diclofenac) or grade-3 (e.g. ibuprofen + ibuprofen) [18]. Even though Kovac et al. investigated the effects of dual use in only one medication class, their results indicate that PIDPs might entail deleterious clinical outcomes.

“Duplicate prescriptions” of inhaled medications have been investigated by Kardos et al. [35]. In contrast to our study, however, their units of analysis were prescriptions in the database of a large Dutch mail order pharmacy [35], and not patients. Meaningful comparisons to our study can therefore not be drawn.

A major strength of our study in comparison with other investigations in the field is that we included two complete calendar years in our analysis, thereby diminishing the effect of seasonal fluctuations. The study by Takahashi et al., by contrast, was conducted during a single winter month and, not surprisingly, showed the highest frequency of “duplicative prescriptions” for “cough and cold drugs” (ATC code R05) [36]. Similarly, the study by Kinoshita et al. was conducted in spring and detected a high rate of duplicative medications for anti-allergic drugs, likely reflecting an increased therapeutic demand for seasonal pollen allergies during the study period [31].

Further strengths of our study are the large number of enrolled patients, the manual assessment of each case by the primary investigators, and the data validation by an interdisciplinary expert panel comprising specialists from clinical pharmacology, internal medicine, geriatric medicine, emergency medicine, anesthesiology, intensive care medicine, neurology, and psychiatry.

The retrospective nature and monocentric setting of our study clearly represent limitations. The study was conducted at a single university hospital, limiting its external validity. However, the internal validity should be comparatively high because we systematically analyzed the medications of all patients presenting to the ED over the course of 2 years, thereby minimizing selection bias. Even though we did not formally evaluate interrater reliability, we are convinced that the application of our new DP categorization markedly reduced subjective bias and terminated the blending of clinically desired and undesired DPs, which was a substantial limitation of previous studies in the field [30–34, 36]. Our DP categorization was specifically devised by physicians for physicians with direct patient contact. It was not designed, however, for patient counseling and/or drug dispensation at pharmacies. Furthermore, there is substantial overlap between the concepts of duplicate prescriptions (appropriate as well as potentially inappropriate) and drug–drug interactions. For the sake of conciseness, we deliberately omitted the aspect of drug–drug interactions from the definitions of ADPs and PIDPs in our DP categorization (Table 1).

More than one-third of the initially screened patients had to be excluded from our study because of incomplete medication records. This may be explained by the fact that the medication documentation was conducted by emergency physicians during clinical routine. Limited time for documentation in the ED may have played a critical role. In addition, an incomplete medication documentation might point to less morbid patients, which might have led to an overestimation of the PIDP prevalence in our study.

Perhaps the most significant limitation of our study is a lack of information about the number of instances in which PIDPs actually entailed clinical consequences, for example in the form of ADRs. This assessment was beyond the scope of a pilot study and should be the subject of future investigations, along with assessments of causality and preventability of ADRs. Our new DP categorization was readily applicable within the setting of the present study. Yet, further studies are needed to fully elucidate its educational, clinical, and scientific potential. Hohl et al. suggested that “the ED should be considered as a place where medication regimens of incoming high-risk patients should be screened systematically for drug-related problems” [37]. We strongly support this idea and propose to include the systematic screening for PIDPs into the medication review in the ED. Physicians should pay special attention to younger patients using non-opioid analgesics and/or inhalatives, as these patients might be particularly prone to PIDPs.

The true clinical implications of our research remain to be established. Notwithstanding, our study opens avenues for further research. We hypothesize that a reduction of PIDPs will lead to a decrease in ADRs. To unravel the clinical significance of PIDPs, a prospective interventional study—preferably within a multicentric and randomized controlled setting—should be conducted in the future.

## Supplementary Information

Below is the link to the electronic supplementary material.

Supplementary file1 (DOCX 26 KB)

Supplementary file2 (DOCX 34 KB)

Supplementary file3 (DOCX 35 KB)

Supplementary file4 (DOCX 26 KB)

## Data Availability

The data that support the findings of this study are available upon reasonable request from the corresponding author.
