# Supplementary material for: Duplicate prescriptions in the emergency department: a retrospective cohort study
Source: Eur J Clin Pharmacol. 2022 Dec 8;79(2):207–17. doi: 10.1007/s00228-022-03436-6 (PMC9734425; doi:10.1007/s00228-022-03436-6)
Supplement: Supplementary file 2 — Supplementary file2 (DOCX 34 KB) [file 228_2022_3436_MOESM2_ESM.docx]

**Supplementary Table 1:** Absolute and relative frequencies of medication classes including individual substances taken in the study population

| **Medication class** | **Substance(s)** | **n** | **%** |
| --- | --- | --- | --- |
| **All medication classes combined** | | **26,402** | **100** |
| ACE inhibitors | Captopril, cilazapril, enalapril, lisinopril, perindopril, quinapril, ramipril | 1221 | 4.6 |
| Angiotensin receptor blockers | Candesartan, eprosartan, irbesartan, losartan, olmesartan, telmisartan, valsartan | 1013 | 3.8 |
| Beta-blockers | Atenolol, bisoprolol, carvedilol, metoprolol, nebivolol, propranolol, sotalol | 1821 | 6.9 |
| Calcium channel blockers | Amlodipine, diltiazem, felodipine, lercanidipine, nifedipine, nisoldipine, nitrendipine, verapamil | 943 | 3.6 |
| Thiazide(-like) diuretics | Bemetizide, bendroflumethiazide, chlorthalidone, hydrochlorothiazide, indapamide, xipamide | 577 | 2.2 |
| Loop diuretics | Furosemide, piretanide, torasemide | 955 | 3.6 |
| Mineralocorticoid receptor antagonists and other diuretics | Amiloride, canrenone, eplerenone, spironolactone, triamterene | 411 | 1.6 |
| Other antihypertensives | Aliskiren, clonidine, dihydralazine, minoxidil, moxonidine, urapidil | 132 | 0.5 |
| Antiarrhythmics | Amiodarone, dronedarone, flecainide, propafenone | 81 | 0.3 |
| Other cardiovascular agents | Beta-acetyldigoxin, digoxin, digitoxin, isosorbide dinitrate, isosorbide mononitrate, ivabradine, molsidomine, nitroglycerin, pentaerythritol tetranitrate, ranolazine, sacubitril | 375 | 1.4 |
| Platelet aggregation inhibitors | Clopidogrel, low-dose acetylsalicylic acid, prasugrel, ticagrelor | 1372 | 5.2 |
| Direct oral anticoagulants | Apixaban, dabigatran, edoxaban, rivaroxaban | 573 | 2.2 |
| Vitamin K antagonists | Acenocoumarol, phenprocoumon, warfarin | 198 | 0.7 |
| Heparin and heparin analogues | Certoparin, dalteparin, enoxaparin, fondaparinux, heparin, nadroparin, tinzaparin | 84 | 0.3 |
| HMG-CoA reductase inhibitors | Atorvastatin, fluvastatin, pravastatin, rosuvastatin, simvastatin | 1331 | 5.0 |
| Other lipid-lowering agents | Alirocumab, bezafibrate, colestyramine, evolocumab, ezetimibe, fenofibrate, gemfibrozil | 181 | 0.7 |
| Short-acting β_2_-adrenergic receptor agonists | Fenoterol, reproterol, salbutamol, terbutaline | 302 | 1.1 |
| Long-acting β_2_-adrenergic receptor agonists | Formoterol, indacaterol, olodaterol, salmeterol, vilanterol | 398 | 1.5 |
| Short-acting muscarinic receptor antagonists | Ipratropium | 78 | 0.3 |
| Long-acting muscarinic receptor antagonists | Aclidinium, glycopyrronium, tiotropium, umeclidinium | 217 | 0.8 |
| Inhaled corticosteroids | Beclomethasone, budesonide, ciclesonide, fluticasone, mometasone | 369 | 1.4 |
| Histamine-1 receptor antagonists | Azelastine, cetirizine, chlorphenamine, clemastine, desloratadine, dimenhydrinate, dimetindene, doxylamine, ebastine, fexofenadine, hydroxyzine, levocetirizine, loratadine, ketotifen, olopatadine, rupatadine | 137 | 0.5 |
| Opioids | Buprenorphine, codeine, dihydrocodeine, fentanyl, hydromorphone, methadone, morphine, oxycodone, pethidine, piritramide, tapentadol, tilidine, tramadol | 550 | 2.1 |
| Non-steroidal anti-inflammatory drugs | Celecoxib, dexketoprofen, diclofenac, etoricoxib, high-dose acetylsalicylic acid, ibuprofen, indomethacin, ketoprofen, ketorolac, meloxicam, naproxen, phenazone, phenylbutazone | 372 | 1.4 |
| Other non-opioid analgesics | Metamizole, paracetamol | 830 | 3.1 |
| Tricyclic antidepressants | Amitriptyline, doxepin, imipramine, opipramol, trimipramine | 164 | 0.6 |
| Tetracyclic antidepressants | Mianserin, mirtazapine | 108 | 0.4 |
| Selective serotonin reuptake inhibitors | Citalopram, fluoxetine, escitalopram, paroxetine, sertraline | 209 | 0.8 |
| Selective serotonin–norepinephrine reuptake inhibitors | Duloxetine, milnacipran, venlafaxine | 63 | 0.2 |
| Other antidepressants | Agomelatine, bupropion, tianeptine, trazodone | 31 | 0.1 |
| First-generation antipsychotics | Benperidol, chlorprothixene, flupentixol, haloperidol, levomepromazine, melperone, perazine, pipamperone, promethazine, prothipendyl, tiapride, zuclopenthixol | 212 | 0.8 |
| Second-generation antipsychotics | Amisulpride, aripiprazole, clozapine, olanzapine, paliperidone, quetiapine, risperidone, sulpiride, ziprasidone | 190 | 0.7 |
| Antiepileptic drugs | Brivaracetam, carbamazepine, eslicarbazepine acetate, gabapentin, lacosamide, lamotrigine, levetiracetam, oxcarbazepine, phenytoin, pregabalin, primidone, topiramate, valproate, vigabatrin, zonisamide | 408 | 1.5 |
| Benzodiazepines and Z-drugs | Alprazolam, bromazepam, clonazepam, diazepam, flunitrazepam, lorazepam, lormetazepam, nitrazepam, oxazepam, temazepam, zolpidem, zopiclone | 249 | 0.9 |
| Antidementia drugs | Donepezil, galantamine, memantine, nicergoline, rivastigmine | 46 | 0.2 |
| Antidiabetics (excluding insulin and insulin analogues) | Dapagliflozin, dulaglutide, empagliflozin, ertugliflozin, glibenclamide, gliclazide, glimepiride, liraglutide, metformin, saxagliptin, sitagliptin, vildagliptin | 605 | 2.3 |
| Vitamins, hormones, enzymes, growth factors, minerals, trace elements | Abiraterone, alfacalcidol, biotin, buserelin, calcidiol, calcitriol, calcium, chlormadinone, cholecalciferol, coenzyme Q10, cyproterone, darbepoetin alfa, dehydroepiandrosterone, desmopressin, desogestrel, diamine oxidase, dienogest, dihydrotachysterol, Dreisavit, drospirenone, dydrogesterone, Equinovo, erythropoietin alfa, erythropoietin beta, erythropoietin zeta, estradiol, estriol, estrogen, ethinylestradiol, etonogestrel, filgrastim, folic acid, glucagon, iodide, insulin aspart, insulin degludec, insulin detemir, insulin glargine, insulin glulisine, insulin levemir, insulin lispro, iron sulfate, isophane insulin, lactase, lanthanum carbonate, leuprorelin, levonorgestrel, levothyroxine, liothyronine, lipoic acid, magnesium, medrogestone, medroxyprogesterone, melatonin, metenolone, methionine, norethisterone, Ocuvite, omega-3 fatty acid, ornithine aspartate, pancreatin, paricalcitol, potassium, progesterone, provitamin A, pyridoxine, regular insulin, selenium, Sitobact, sodium chloride, testosterone, thiamine, trenbolone, Tromcardin, vitamin B_12_, vitamin C, vitamin E, vitamin K, zinc | 3441 | 13.0 |
| Proton pump inhibitors | Esomeprazole, lansoprazole, omeprazole, pantoprazole, rabeprazole | 1656 | 6.3 |
| Other acid-blocking agents | Aluminium hydroxide, hydrotalcite, magaldrate, magnesium hydroxide, pirenzepine, ranitidine, sodium bicarbonate, sucralfate | 56 | 0.2 |
| Prokinetics | Domperidone, metoclopramide | 108 | 0.4 |
| Laxatives | Bisacodyl, disodium phosphate, lactulose, macrogol 3350, *Plantago ovata*, sodium picosulfate, trisodium citrate | 366 | 1.4 |
| Anti-diarrhoeal drugs | Loperamide | 47 | 0.2 |
| Alpha-blockers | Alfuzosin, doxazosin, tamsulosin, terazosin, silodosin | 239 | 0.9 |
| Urinary antispasmodics | Darifenacin, fesoterodine, oxybutynin, propiverine, tolterodine, trospium, solifenacin | 49 | 0.2 |
| 5-alpha reductase inhibitors | Dutasteride, finasteride | 27 | 0.1 |
| Immunosuppressants and immune modulators | Adalimumab, anakinra, apremilast, azathioprine, belatacept, belimumab, brodalumab, canakinumab, ciclosporin, cloprednol, cortisone, cromoglicic acid, dexamethasone, eculizumab, etanercept, etrolizumab, everolimus, fingolimod, glatiramer acetate, hydrocortisone, hydroxychloroquine, infliximab, interferon beta, leflunomide, lenalidomide, mesalazine, montelukast, mycophenolate mofetil, mycophenolic acid, natalizumab, ocrelizumab, omalizumab, oxaceprol, pimecrolimus, pomalidomide, prednisolone, prednisone, secukinumab, sirolimus, sulfasalazine, tacrolimus, teriflunomide, tofacitinib, ustekinumab, vedolizumab | 1041 | 3.9 |
| Antineoplastic agents | Alectinib, anastrozole, atezolizumab, bevacizumab, bicalutamide, cabozantinib, capecitabine, carboplatin, cisplatin, cyclophosphamide, dabrafenib, dasatinib, doxorubicin, enzalutamide, epirubicin, exemestane, fluorouracil, fulvestrant, gemcitabine, gilteritinib, hydroxycarbamide, ibrutinib, imatinib, invatinib, lanreotide, lenvatinib, letrozole, melphalan, mercaptopurine, methotrexate, nilotinib, nintedanib, nivolumab, olaratumab, osimertinib, paclitaxel, palbociclib, pembrolizumab, pemetrexed, rituximab, ruxolitinib, sunitinib, tamoxifen, trabectedin, trametinib, venetoclax | 158 | 0.6 |
| Antibiotics | Amoxicillin, ampicillin, azithromycin, benzylpenicillin, cefaclor, cefadroxil, cefpodoxime, ceftriaxone, cefuroxime, ciprofloxacin, clarithromycin, clavulanic acid, clindamycin, colistin, dapsone, doxycycline, ethambutol, fosfomycin, fusidic acid, gentamicin, isoniazid, levofloxacin, linezolid, meropenem, metronidazole, minocycline, moxifloxacin, mupirocin, neomycin, nitrofurantoin, nitrofurazone, nitroxoline, norfloxacin, ofloxacin, penicillin, pyrazinamide, rifabutin, rifampicin, rifaximin, roxithromycin, sulbactam, sulfamethoxazole, sultamicillin, tobramycin, trimethoprim | 585 | 2.2 |
| Antimycotics | Amphotericin B, fluconazole, itraconazole, nystatin, posaconazole, voriconazole | 88 | 0.3 |
| Antivirals | Abacavir, aciclovir, amantadine, bictegravir, brivudine, darunavir, dolutegravir, elvitegravir, emtricitabine, entecavir, lamivudine, nevirapine, oseltamivir, raltegravir, ritonavir, sofosbuvir, tenofovir, valganciclovir, velpatasvir | 135 | 0.5 |
| Topical ophthalmics | Acetazolamide, betaxolol, bimatoprost, brimonidine, brinzolamide, dorzolamide, ectoine, hydroxypropyl guar, hyaluronic acid, hypromellose, latanoprost, levobunolol, monoprost, pilocarpine, polyacrylic acid, Systane, tafluprost, timolol, travoprost | 139 | 0.5 |
| Topical dermatics | Betamethasone, bifonazol, Cavilon, ciclopirox, clioquinol, clotrimazole, dexpanthenol, hydrocortisone, isotretinoin, miconazole, Nisita, phenolsulfonic acid, prednicarbate, tretinoin, urea | 47 | 0.2 |
| Bisphosphonates | Alendronic acid, ibandronic acid, pamidronic acid, risedronic acid | 52 | 0.2 |
| Antiparkinson drugs | Benserazide, biperiden, bornaprine, bromocriptine, budipine, cabergoline, carbidopa, entacapone, levodopa, piribedil, pramipexole, rasagiline, ropinirole, rotigotine, safinamide, trihexyphenidyl | 252 | 1.0 |
| Muscle relaxants | Baclofen, dantrolene, methocarbamol, tizanidine, tolperisone | 56 | 0.2 |
| Phytopharmaceuticals | Algae, *Aesculus hippocastanum*, Aplona, Augen Fit, BactoFlor, Bronchipret, camphor, Canephron, Cardiodoron, *Carum carvi*, *Cassia senna*, *Colchicum autumnale*, cranberry, *Crataegus*, eucalyptol, frankincense, Gaviscon, Gelomyrtol, *Ginkgo biloba*, *Hamamelis*, *Harpagophytum procumbens*, *Hedera helix*, Iberogast, lavender oil, nattokinase, Neurexan, *Passitrabecflora*, Padma28active, peppermint extract, resveratrol, saw palmetto, Sinupret, *Spirulina*, St. John’s wort, *Strychnos nux-vomica*, Syntrival, *Thymus vulgaris*, turmeric, *Urtica*, *Valeriana*, *Viscum*, *Vitex agnus-castus* | 121 | 0.5 |
| Homoeopathic preparations | Bach flower solution, Synergon 6 Sepia, Vertigoheel | 3 | < 0.1 |
| Miscellaneous | Acitretin, amfepramone, 2-aminoethyl dihydrogen phosphate, acetylcysteine, activated carbon, albendazole, allopurinol, ambroxol, aprepitant, arginine, atomoxetine, atovaquone, benzbromarone, benzydamine, betahistine, bethanechol, *Bifidobacterium longum*, buspirone, caffeine, carbimazole, chlorhexidine, cilostazol, cinacalcet, cinnarizine, cobicistat, colchicine, deferiprone, denosumab, dextromethorphane, dimethicone, dornase alfa, drotaverine, eltrombopag, erenumab, *Escherichia coli* Nissle 1917, etelcalcetide, fampridine, febuxostat, fludrocortisone, flunarizine, Fresubin, Gelclair, granisetron, hyoscine butylbromide, iloprost, ivacaftor, ivermectin, *Lactobacillus acidophilus*, lidocaine, lithium, lumacaftor, macitentan, mannose, mebeverine, mesna, methylphenidate, midodrine, mirabegron, modafinil, naloxone, naratriptan, nitazoxanide, noscapine, octenidine dihydrochloride, octocog alfa, octreotide, ondansetron, palonosetron, patiromer, pentoxifylline, pentoxyverine, perchlorate, proguanil, propylthiouracil, pseudoephedrine, pyridostigmine, quinine, riluzole, riociguat, rizatriptan, roflumilast, *Sacharomyces boulardii*, scopolamine, sevelamer, sildenafil, silymarin, simeticone, strontium ranelate, sumatriptan, Symbioflor, tadalafil, tafamidis, tetrabenazine, tetrahydrocannabinol, theophylline, thiamazol, tolevamer, tolvaptan, tranexamic acid, treprostinil, tyloxapol, ursodeoxycholic acid, vonicog alfa, whale oil, xylometazoline | 930 | 3.5 |

ACE denotes angiotensin-converting enzyme, HMG-CoA hydroxymethylglutaryl coenzyme A.
