# Supplementary material for: Duplicate prescriptions in the emergency department: a retrospective cohort study
Source: Eur J Clin Pharmacol. 2022 Dec 8;79(2):207–17. doi: 10.1007/s00228-022-03436-6 (PMC9734425; doi:10.1007/s00228-022-03436-6)
Supplement: Supplementary file 3 — Supplementary file3 (DOCX 35 KB) [file 228_2022_3436_MOESM3_ESM.docx]

**Supplementary Table 2:** Absolute and relative frequencies of potentially inappropriate duplicate prescriptions detected in the study population

| **PIDPs** | **n** | **%**^a^ |
| --- | --- | --- |
| **Grade-1 PIDPs** | **263** | **100** |
| **Non-opioid analgesics** | **139** | **52.9** |
| Acetylsalicylic acid + diclofenac | 1 | 0.4 |
| Acetylsalicylic acid + paracetamol | 7 | 2.7 |
| Celecoxib + metamizole | 5 | 1.9 |
| Dexketoprofen + metamizole | 1 | 0.4 |
| Diclofenac + metamizole | 2 | 0.8 |
| Diclofenac + paracetamol | 2 | 0.8 |
| Etoricoxib + ibuprofen | 1 | 0.4 |
| Etoricoxib + metamizole | 12 | 4.6 |
| Ibuprofen + metamizole | 40 | 15.2 |
| Ibuprofen + paracetamol | 27 | 10.3 |
| Metamizole + naproxen | 1 | 0.4 |
| Metamizole +NSAID (not otherwise specified) | 1 | 0.4 |
| Metamizole + paracetamol | 37 | 14.1 |
| Metamizole + phenylbutazone | 1 | 0.4 |
| Naproxen + paracetamol | 1 | 0.4 |
| **Opioids** | **6** | **2.3** |
| Fentanyl + loperamide | 2 | 0.8 |
| Hydromorphone + loperamide | 1 | 0.4 |
| Loperamide + tapentadol | 1 | 0.4 |
| Loperamide + tilidine | 2 | 0.8 |
| **Antidepressants** | **19** | **7.2** |
| Amitriptyline + agomelatine | 1 | 0.4 |
| Amitriptyline + duloxetine | 2 | 0.8 |
| Amitriptyline + escitalopram | 1 | 0.4 |
| Amitriptyline + fluoxetine | 1 | 0.4 |
| Amitriptyline + mirtazapine | 2 | 0.8 |
| Amitriptyline + venlafaxine | 1 | 0.4 |
| Bupropion + doxepin | 1 | 0.4 |
| Citalopram + mirtazapine | 1 | 0.4 |
| Citalopram + trimipramine | 1 | 0.4 |
| Escitalopram + trimipramine | 4 | 1.5 |
| Mirtazapine + trimipramine | 1 | 0.4 |
| Opipramol + sertraline | 1 | 0.4 |
| Opipramol + trimipramine | 1 | 0.4 |
| Opipramol + venlafaxine | 1 | 0.4 |
| **Antipsychotics** | **9** | **3.4** |
| Aripiprazole + olanzapine | 1 | 0.4 |
| Amisulpride + flupentixol | 1 | 0.4 |
| Chlorprothixene + haloperidol | 1 | 0.4 |
| Chlorprothixene + olanzapine | 1 | 0.4 |
| Chlorprothixene + quetiapine | 2 | 0.8 |
| Flupentixol +risperidone | 1 | 0.4 |
| Haloperidol + olanzapine | 2 | 0.8 |
| **Sedatives** | **28** | **10.6** |
| Alprazolam + zopiclone | 1 | 0.4 |
| Amitriptyline + pipamperone | 3 | 1.1 |
| Bromazepam + quetiapine | 1 | 0.4 |
| Diazepam + melperone | 1 | 0.4 |
| Diazepam + zolpidem | 1 | 0.4 |
| Lorazepam + melperone | 1 | 0.4 |
| Lorazepam + mirtazapine | 1 | 0.4 |
| Lorazepam + pipamperone | 4 | 1.5 |
| Lorazepam + promethazine | 1 | 0.4 |
| Lorazepam + zolpidem | 2 | 0.8 |
| Melperone + mirtazapine | 1 | 0.4 |
| Melperone + quetiapine | 2 | 0.8 |
| Melperone + trimipramine | 1 | 0.4 |
| Oxazepam + pipamperone | 1 | 0.4 |
| Oxazepam + zopiclone | 1 | 0.4 |
| Pipamperone + promethazine | 1 | 0.4 |
| Pipamperone + quetiapine | 3 | 1.1 |
| Promethazine + quetiapine | 1 | 0.4 |
| Trimipramine + zopiclone | 1 | 0.4 |
| **Antiepileptic drugs** | **6** | **2.3** |
| Carbamazepine + valproate | 1 | 0.4 |
| Lamotrigine + pregabalin | 1 | 0.4 |
| Lamotrigine + oxcarbazepine | 1 | 0.4 |
| Lamotrigine + topiramate | 1 | 0.4 |
| Levetiracetam + valproate | 1 | 0.4 |
| Pregabalin + valproate | 1 | 0.4 |
| **Platelet aggregation inhibitors and anticoagulants** | **17** | **6.5** |
| Apixaban + clopidogrel | 3 | 1.1 |
| Apixaban + low-dose acetylsalicylic acid | 3 | 1.1 |
| Clopidogrel + low-dose acetylsalicylic acid | 2 | 0.8 |
| Clopidogrel + phenprocoumon | 1 | 0.4 |
| Clopidogrel + rivaroxaban | 1 | 0.4 |
| Dabigatran + low-dose acetylsalicylic acid | 1 | 0.4 |
| Enoxaparin + phenprocoumon | 1 | 0.4 |
| Fondaparinux + low-dose acetylsalicylic acid | 1 | 0.4 |
| Low-dose acetylsalicylic acid + phenprocoumon | 1 | 0.4 |
| Low-dose acetylsalicylic acid + rivaroxaban | 2 | 0.8 |
| Prasugrel + rivaroxaban | 1 | 0.4 |
| **RAAS inhibitors** | **7** | **2.7** |
| Candesartan + ramipril | 2 | 0.8 |
| Enalapril + losartan | 1 | 0.4 |
| Enalapril + valsartan | 2 | 0.8 |
| Irbesartan + ramipril | 1 | 0.4 |
| Ramipril + valsartan | 1 | 0.4 |
| **Calcium channel blockers** | **4** | **1.5** |
| Amlodipine + verapamil | 2 | 0.8 |
| Lercanidipine + verapamil | 1 | 0.4 |
| Nifedipine + verapamil | 1 | 0.4 |
| **Acid-blocking agents** | **7** | **2.7** |
| Omeprazole + ranitidine | 1 | 0.4 |
| Omeprazole + sucralfate | 1 | 0.4 |
| Pantoprazole + hydrotalcite | 1 | 0.4 |
| Pantoprazole + ranitidine | 1 | 0.4 |
| Pantoprazole + sucralfate | 1 | 0.4 |
| PPI (not otherwise specified) + aluminium hydroxide/magnesium hydroxide | 1 | 0.4 |
| Ranitidine + sodium hydrogen carbonate | 1 | 0.4 |
| **Miscellaneous** | **21** | **8.0** |
| Amphotericin B + nystatin | 1 | 0.4 |
| Atorvastatin + fenofibrate | 1 | 0.4 |
| Baclofen + tizanidine | 1 | 0.4 |
| Beclomethasone dipropionate + prednisolone | 2 | 0.8 |
| β-Acetyldigoxin + bisoprolol | 1 | 0.4 |
| Bisoprolol + propranolol | 1 | 0.4 |
| Budesonide + prednisolone | 4 | 1.5 |
| Buspirone + escitalopram | 1 | 0.4 |
| Clonidine + moxonidine | 2 | 0.8 |
| Fluticasone furoate + prednisolone | 1 | 0.4 |
| Fluticasone propionate + prednisolone | 1 | 0.4 |
| Gemfibrozil + simvastatin | 1 | 0.4 |
| Lactulose + macrogol 3350 | 1 | 0.4 |
| Methocarbamol + tolperisone | 1 | 0.4 |
| Noscapine + pentoxyverine | 1 | 0.4 |
| Testosterone + trenbolone | 1 | 0.4 |
| **Grade-2 PIDPs** | **215** | **100** |
| **Non-opioid analgesics** | **14** | **6.5** |
| Acetylsalicylic acid + diclofenac | 1 | 0.5 |
| Acetylsalicylic acid + ibuprofen | 2 | 0.9 |
| Diclofenac + etoricoxib | 1 | 0.5 |
| Diclofenac + ibuprofen | 9 | 4.2 |
| Etoricoxib + ibuprofen | 1 | 0.5 |
| **Opioids** | **38** | **17.7** |
| Buprenorphine + codeine | 1 | 0.5 |
| Buprenorphine + dihydrocodeine | 1 | 0.5 |
| Buprenorphine + fentanyl | 2 | 0.9 |
| Buprenorphine + oxycodone | 1 | 0.5 |
| Buprenorphine + tilidine | 1 | 0.5 |
| Codeine + fentanyl | 1 | 0.5 |
| Codeine + tilidine | 2 | 0.9 |
| Dihydrocodeine + hydromorphone | 1 | 0.5 |
| Fentanyl + hydromorphone | 1 | 0.5 |
| Fentanyl + morphine | 8 | 3.7 |
| Fentanyl + oxycodone | 4 | 1.9 |
| Fentanyl + piritramide | 3 | 1.4 |
| Hydromorphone + morphine | 1 | 0.5 |
| Hydromorphone + tilidine | 1 | 0.5 |
| Hydromorphone + tramadol | 1 | 0.5 |
| Methadone + oxycodone | 2 | 0.9 |
| Morphine + tilidine | 1 | 0.5 |
| Oxycodone + tilidine | 1 | 0.5 |
| Oxycodone + tramadol | 1 | 0.5 |
| Pethidine + tilidine | 2 | 0.9 |
| Tapentadol + tramadol | 1 | 0.5 |
| Tilidine + tramadol | 1 | 0.5 |
| **Antipsychotics** | **10** | **4.7** |
| Amisulpride + quetiapine | 3 | 1.4 |
| Chlorprothixene + haloperidol | 2 | 0.9 |
| Chlorprothixene + levomepromazine | 1 | 0.5 |
| Chlorprothixene + melperone | 1 | 0.5 |
| Chlorprothixene + promethazine | 1 | 0.5 |
| Flupentixol + haloperidol | 1 | 0.5 |
| Flupentixol + perazine | 1 | 0.5 |
| **Benzodiazepines** | **10** | **4.7** |
| Alprazolam + diazepam | 1 | 0.5 |
| Alprazolam + lorazepam | 1 | 0.5 |
| Bromazepam + diazepam | 2 | 0.9 |
| Clonazepam + lorazepam | 1 | 0.5 |
| Diazepam + lorazepam | 3 | 1.4 |
| Lorazepam + oxazepam | 1 | 0.5 |
| Lorazepam + temazepam | 1 | 0.5 |
| **Other sedatives** | **8** | **3.7** |
| Melperone + pipamperone | 2 | 0.9 |
| Melperone + promethazine | 1 | 0.5 |
| Perazine + promethazine | 1 | 0.5 |
| Promethazine + prothipendyl | 1 | 0.5 |
| Quetiapine + risperidone | 3 | 1.4 |
| **Inhalatives** | **75** | **34.9** |
| Fenoterol + formoterol | 5 | 2.3 |
| Fenoterol + indacaterol | 2 | 0.9 |
| Fenoterol + olodaterol | 1 | 0.5 |
| Fenoterol + reproterol | 1 | 0.5 |
| Fenoterol + salbutamol | 11 | 5.1 |
| Fenoterol + salmeterol | 2 | 0.9 |
| Fenoterol + terbutaline | 1 | 0.5 |
| Fenoterol + vilanterol | 1 | 0.5 |
| Formoterol + indacaterol | 5 | 2.3 |
| Formoterol + olodaterol | 2 | 0.9 |
| Formoterol + reproterol | 1 | 0.5 |
| Formoterol + salbutamol | 9 | 4.2 |
| Formoterol + terbutaline | 1 | 0.5 |
| Formoterol + vilanterol | 1 | 0.5 |
| Indacaterol + olodaterol | 1 | 0.5 |
| Indacaterol + salbutamol | 4 | 1.9 |
| Olodaterol + salbutamol | 1 | 0.5 |
| Salbutamol + salmeterol | 1 | 0.5 |
| Salbutamol + vilanterol | 3 | 1.4 |
| Glycopyrronium bromide + ipratropium bromide | 8 | 3.7 |
| Glycopyrronium bromide + tiotropium bromide | 2 | 0.9 |
| Ipratropium bromide + tiotropium bromide | 4 | 1.9 |
| Ipratropium bromide + umeclidinium bromide | 1 | 0.5 |
| Beclomethasone dipropionate + budesonide | 3 | 1.4 |
| Beclomethasone dipropionate + fluticasone furoate | 3 | 1.4 |
| Fluticasone (not otherwise specified) + fluticasone furoate | 1 | 0.5 |
| **RAAS inhibitors** | **3** | **1.4** |
| Candesartan + valsartan | 1 | 0.5 |
| Captopril + ramipril | 1 | 0.5 |
| Perindopril + ramipril | 1 | 0.5 |
| **Calcium channel blockers** | **17** | **7.9** |
| Amlodipine + lercanidipine | 2 | 0.9 |
| Amlodipine + nifedipine | 6 | 2.8 |
| Amlodipine + nitrendipine | 6 | 2.8 |
| Felodipine + nifedipine | 1 | 0.5 |
| Lercanidipine + nifedipine | 1 | 0.5 |
| Nifedipine + nitrendipine | 1 | 0.5 |
| **Diuretics** | **11** | **5.1** |
| Chlorthalidone + hydrochlorothiazide | 1 | 0.5 |
| Hydrochlorothiazide + indapamide | 4 | 1.9 |
| Furosemide + piritanide | 1 | 0.5 |
| Furosemide + torasemide | 5 | 2.3 |
| **α_1_-Adrenergic receptor antagonists** | **3** | **1.4** |
| Alfuzosin + silodosin | 1 | 0.5 |
| Alfuzosin + tamsulosin | 2 | 0.9 |
| **Miscellaneous** | **25** | **11.6** |
| Allopurinol + febuxostat | 1 | 0.5 |
| Atorvastatin + simvastatin | 2 | 0.9 |
| Benserazide + carbidopa | 2 | 0.9 |
| Benserazide + peripheral dopa-decarboxylase inhibitor (not otherwise specified) | 1 | 0.5 |
| Bisacodyl + sodium picosulfate | 3 | 1.4 |
| Clemastine + desloratadine | 2 | 0.9 |
| Clotrimazole + miconazole | 1 | 0.5 |
| Darifenacin + tolterodine | 1 | 0.5 |
| Escitalopram + sertraline | 1 | 0.5 |
| Gabapentin + pregabalin | 1 | 0.5 |
| Hydrocortisone + prednisolone | 2 | 0.9 |
| Isosorbide dinitrate + pentaerythritol tetranitrate | 2 | 0.9 |
| Levothyroxine + liothyronine | 2 | 0.9 |
| Omeprazole + pantoprazole | 1 | 0.5 |
| Ondansetron + palonosetron | 1 | 0.5 |
| Phenprocoumon + dalteparin | 1 | 0.5 |
| Phenprocoumon + tinzaparin | 1 | 0.5 |
| Rupatadine + desloratadine | 1 | 0.5 |
| **Grade-3 PIDPs** | **49** | **100** |
| **Non-opioid analgesics** | **2** | **4.1** |
| Ibuprofen + ibuprofen | 1 | 2.0 |
| Metamizole + metamizole | 1 | 2.0 |
| **Opioids** | **3** | **6.1** |
| Tapentadol + tapentadol | 2 | 4.1 |
| Tilidine + tilidine | 1 | 2.0 |
| **Inhalatives** | **11** | **22.4** |
| Budesonide + budesonide | 2 | 4.1 |
| Formoterol + formoterol | 1 | 2.0 |
| Glycopyrronium bromide + glycopyrronium bromide | 1 | 2.0 |
| Indacaterol + indacaterol | 1 | 2.0 |
| Ipratropium bromide + ipratropium bromide | 2 | 4.1 |
| Salbutamol + salbutamol | 4 | 8.2 |
| **RAAS inhibitors** | **3** | **6.1** |
| Candesartan + candesartan | 1 | 2.0 |
| Ramipril + ramipril | 2 | 4.1 |
| **Calcium channel blockers** | **2** | **4.1** |
| Amlodipine + amlodipine | 1 | 2.0 |
| Lercanidipine + lercanidipine | 1 | 2.0 |
| **Diuretics** | **2** | **4.1** |
| Hydrochlorothiazide + hydrochlorothiazide | 1 | 2.0 |
| Torasemide + torasemide | 1 | 2.0 |
| **Minerals** | **5** | **10.2** |
| Calcium + calcium | 1 | 2.0 |
| Magnesium + magnesium | 3 | 6.1 |
| Potassium + potassium | 1 | 2.0 |
| **Miscellaneous** | **21** | **42.9** |
| Acetylsalicylic acid + acetylsalicylic acid | 1 | 2.0 |
| Atorvastatin + atorvastatin | 1 | 2.0 |
| Cholecalciferol + cholecalciferol | 7 | 14.3 |
| Clemastine + clemastine | 1 | 2.0 |
| Domperidone + domperidone | 1 | 2.0 |
| Hyaluronic acid + hyaluronic acid | 1 | 2.0 |
| Insulin aspart + insulin aspart | 1 | 2.0 |
| Levodopa + levodopa | 1 | 2.0 |
| Lidocaine + lidocaine | 1 | 2.0 |
| Metoprolol + metoprolol | 1 | 2.0 |
| Moxonidine + moxonidine | 1 | 2.0 |
| Naloxone + naloxone | 2 | 4.1 |
| Olanzapine + olanzapine | 1 | 2.0 |
| Ondansetron + ondansetron | 1 | 2.0 |

^a^Percentages may not total 100 because of rounding.

NSAID denotes nonsteroidal anti-inflammatory drug, PIDP potentially inappropriate duplicate prescription, PPI proton pump inhibitor, and RAAS renin–angiotensin–aldosterone system.
