# Supplementary material for: Duplicate prescriptions in the emergency department: a retrospective cohort study
Source: Eur J Clin Pharmacol. 2022 Dec 8;79(2):207–17. doi: 10.1007/s00228-022-03436-6 (PMC9734425; doi:10.1007/s00228-022-03436-6)
Supplement: Supplementary file 4 — Supplementary file4 (DOCX 26 KB) [file 228_2022_3436_MOESM4_ESM.docx]

| **Supplementary Table 3A:** Number of patients affected by at least one grade-1 potentially inappropriate duplicate prescription, stratified by age groups and medication classes involved | | | | | | | | | | | | |
| --- | --- | --- | --- | --- | --- | --- | --- | --- | --- | --- | --- | --- |
| **Age group (years)** | **Non-opioid analge­sics** | **Opioids** | **Anti­depressants** | **Anti­psychotics** | **Sedatives** | **Antiepi­leptic drugs** | **PAIs and anticoagu­lants** | **RAAS inhibitors** | **Calcium channel blockers** | **Acid-blocking agents** | **Miscella­neous** | **Total count** |
| 18–29 | 27 | – | – | 1 | 3 | 1 | – | 2 | – | – | 1 | **35** |
| 30–39 | 17 | – | – | 2 | 2 | 2 | – | – | – | – | 1 | **24** |
| 40–49 | 16 | – | 4 | 2 | 1 | – | 1 | 3 | – | 2 | 3 | **32** |
| 50–59 | 23 | 2 | 8 | 1 | 4 | – | – | – | – | 1 | 5 | **44** |
| 60–69 | 17 | – | 2 | – | 3 | 1 | 2 | 1 | 2 | – | 4 | **32** |
| 70–79 | 20 | 3 | 1 | 1 | 6 | 2 | 8 | – | 1 | 2 | 2 | **46** |
| 80–89 | 16 | – | 3 | 1 | 8 | – | 4 | 1 | 1 | – | 1 | **35** |
| ≥ 90 | 4 | 1 | – | – | 1 | – | 2 | – | – | 1 | 1 | **10** |
| **Total count** | **140** | **6** | **18** | **8** | **28** | **6** | **17** | **7** | **4** | **6** | **18** | **258** |

PAI denotes platelet aggregation inhibitor, RAAS renin–angiotensin–aldosterone system.

| **Supplementary Table 3B:** Number of patients affected by at least one grade-2 potentially inappropriate duplicate prescription, stratified by age groups and medication classes involved | | | | | | | | | | | |
| --- | --- | --- | --- | --- | --- | --- | --- | --- | --- | --- | --- |
| **Age group (years)** | **Non-opioid analgesics** | **Opioids** | **Antipsychotics** | **Benzodiazepines** | **Sedatives** | **Inhalatives** | **RAAS inhibitors** | **Calcium channel blockers** | **Diuretics** | **Miscellaneous** | **Total count** |
| 18–29 | 2 | 6 | 1 | – | 1 | 6 | – | 1 | – | 2 | **19** |
| 30–39 | 2 | 3 | 2 | – | – | 4 | – | 1 | – | 3 | **15** |
| 40–49 | 1 | 6 | 3 | 1 | 1 | 7 | – | – | 2 | 2 | **23** |
| 50–59 | 5 | 5 | – | 5 | 1 | 4 | – | 3 | 3 | 4 | **30** |
| 60–69 | – | 9 | 4 | 1 | – | 11 | – | 2 | 4 | 3 | **34** |
| 70–79 | 2 | 5 | – | – | 1 | 19 | 2 | 7 | 1 | 5 | **42** |
| 80–89 | 2 | 3 | – | 1 | 1 | 6 | – | 1 | – | 5 | **19** |
| ≥ 90 | – | 1 | – | – | 2 | 2 | – | 2 | – | 1 | **8** |
| **Total count** | **14** | **38** | **10** | **8** | **7** | **59** | **2** | **17** | **10** | **25** | **190** |

RAAS denotes renin–angiotensin–aldosterone system.

| **Supplementary Table 3C:** Number of patients affected by at least one grade-3 potentially inappropriate duplicate prescription, stratified by age groups and medication classes involved | | | | | | | | | |
| --- | --- | --- | --- | --- | --- | --- | --- | --- | --- |
| **Age group (years)** | **Non-opioid analgesics** | **Opioids** | **Inhalatives** | **RAAS inhibitors** | **Calcium channel blockers** | **Diuretics** | **Minerals** | **Miscellaneous** | **Total count** |
| 18–29 | – | 1 | – | – | – | – | – | 1 | **2** |
| 30–39 | – | – | – | – | – | – | 3 | 1 | **4** |
| 40–49 | – | 1 | 3 | – | 2 | – | – | 2 | **8** |
| 50–59 | 1 | – | 2 | – | – | – | 1 | 3 | **7** |
| 60–69 | – | – | 2 | 1 | – | – | – | 3 | **6** |
| 70–79 | – | 1 | 2 | 2 | – | 1 | – | 4 | **10** |
| 80–89 | – | 1 | – | – | – | – | – | 3 | **4** |
| ≥ 90 | 1 | – | 1 | – | 1 | 1 | – | – | **4** |
| **Total count** | **2** | **4** | **10** | **3** | **3** | **2** | **4** | **17** | **45** |

RAAS denotes renin–angiotensin–aldosterone system.
